# Romanticism at the Edge of Death

**DOI:** 10.3201/eid3203.AC3203

**Published:** 2026-03

**Authors:** Terence Chorba

**Affiliations:** Centers for Disease Control and Prevention, Atlanta, Georgia, USA

**Keywords:** tuberculosis and other mycobacteria, bacteria, John Keats, Joseph Severn, Portrait of Keats listening to a nightingale on Hampstead Heath, art–science connection

**Figure 1:**
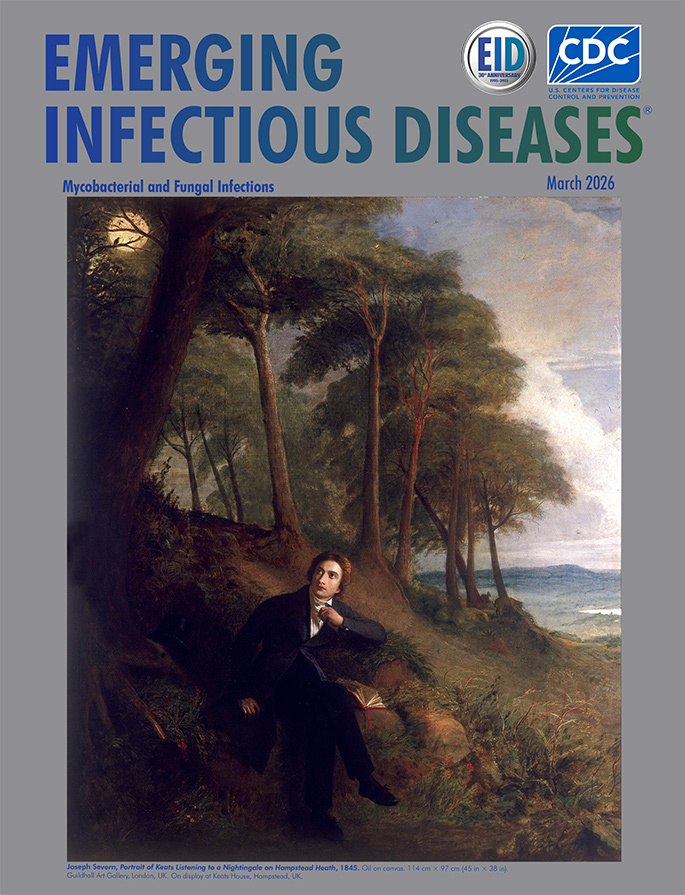
**Joseph Severn, *Portrait of Keats, listening to a nightingale on Hampstead Heath*, 1845.** Oil on canvas. 114 cm × 97 cm (45 in × 38 in). Guildhall Art Gallery, London, UK. On display at Keats House, Hampstead, UK.

Fade softly from my eyes, and be once more  In masque-like figures on the dreary urn; Farewell! I yet have visions for the night,  And for the day faint visions there is store;  Vanish, ye Phantoms! from my idle spright, Into the clouds, and never more return! —John Keats, *Ode on Indolence*, lines 55–60 (*1*)

John Keats (1795–1821), a renowned poet of the English Romantic movement, died at just 25 years of age, a victim of what was then called consumption; in 1829, Johann Schonlein, a German physician, renamed the disease tuberculosis (*2*). As a young man, Keats was apprenticed to an apothecary and trained to be a physician at Guy’s Hospital in London, obtaining the Licentiate of the Society of Apothecaries in 1816, which made him eligible to practice both pharmacy and medicine (*3*). Although he achieved the rank of assistant surgeon at age 20, Keats was enamored of the beauty of poetry and truncated his medical practice.

The severity of Keats’ illness was presaged by the deaths from tuberculosis of his mother when he was 14 (*4*) and of a younger brother, Thomas (1799–1818), 3 years before his own death (*5*). His illness deeply shaped his creative output and posthumous legacy. Students of English poetry are awed when they learn how, in a single year, 1819, Keats composed 6 elaborately structured odes (*Ode on a Grecian Urn*, *Ode on Indolence*, *Ode on Melancholy*, *Ode to a Nightingale*, *Ode to Psyche*, and *To Autumn*) that rank among his best known poems, together with numerous sonnets and other works. Although not widely known in his brief lifetime but well launched toward literary immortality, Keats had to confront the reality of his own impending death from tuberculosis when, in February 1820, he coughed up blood and noted (as a physician) that it “was arterial”—“my death warrant” (*6*).

In early 19th Century Europe, Keats’ disease would have been interpreted through a dominant framework of heredity, because the disease was so often observed in the context of families who had shared airspace (*7*). In 1865, by demonstrating that inoculation with tuberculin material from human lymph nodes would result in the development of tubercular lesions in rabbits, Jean-Antoine Villemin provided evidence to the scientific community that tuberculosis was infectious, a position that largely went ignored (*8*). At the time, in the absence of common knowledge of a communicable etiology and of a known cure, a common prescription for consumption was a change of climate. That was before the definitive breakthrough revelation by Robert Koch on March 24, 1882, that the etiology of this disease was infection with a *Mycobacterium* (*9*); the date has been designated as World TB Day by the World Health Organization and is why March issues of *Emerging Infectious Diseases* always have tuberculosis as their central and cover themes. 

For Keats, a change of climate meant setting sail for Italy in September 1820, accompanied by his friend Joseph Severn (1793–1879), in the hopes that the mild Mediterranean air would ease his suffering. Severn was an English portrait painter who had developed a reputation as a good miniaturist, and for his artwork, he won a medal and 3-year traveling fellowship from the Royal Academy. It is thought that Severn had first met Keats in 1816, and in London’s Royal Academy Exhibition of 1819, Severn exhibited a portrait miniature, *J. Keats, Esq.* (Figure). Although Severn had an exceedingly successful artistic career as a realist and genre painter after Keats’ death, helped establish the British Academy of Arts in Rome, and later served as British consul (1861–1872) in Rome at the time of Italian unification under Giuseppi Garibaldi, he is best known for his role in nursing Keats through his final days and for the several artistic depictions that he made of the poet. On this issue’s cover is a depiction of the poet painted by Severn in 1845, *Keats Listening to a Nightingale on Hampstead Heath.* In this posthumous portrait, Severn pictures a distracted Keats seated in an environment that is at once day and night, two scenes in one, with his book set aside, the poet gazing toward a small bird silhouetted against a full moon.

**Figure 2:**
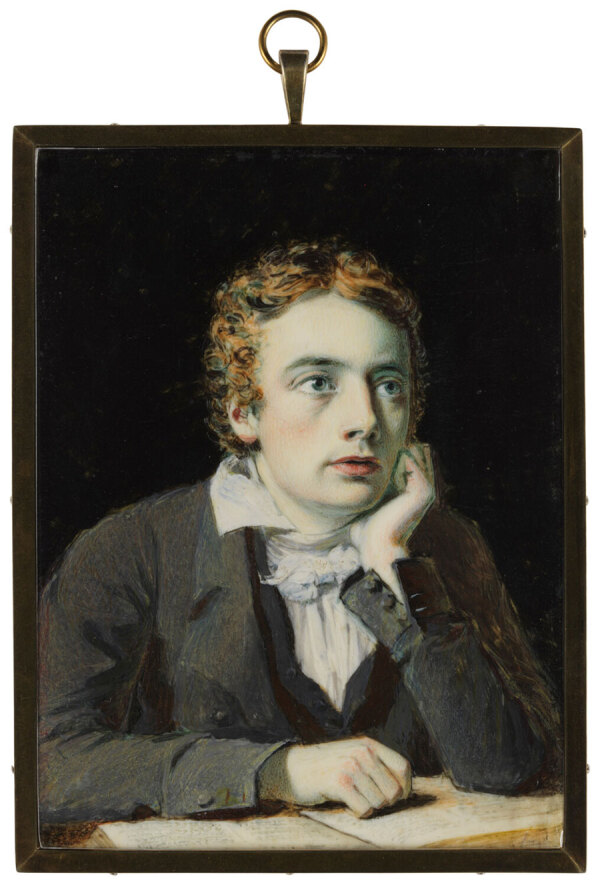
Joseph Severn, *John Keats, Esq.*, 1819. Oil on ivory miniature. 105 × 79 mm. National Portrait Gallery, London, UK. https://www.npg.org.uk/collections/search/portrait/mw03554/John-Keats

After arriving in Rome in November 1820 enduring rapid disease progression, and after numerous contemporary medical interventions including starvation and bloodletting, Keats died in Rome in February 1821 (*10*). Soon thereafter, his room and its contents were burned, the walls scraped, and the apartment cleansed, all consistent with the then-still-unproven theory that consumption might be infectious in origin (*11*).

Because tuberculosis was so widespread in 19th Century Europe populations, accounting for estimates of 1 in 4 deaths at the time (*12*), the presence of many young, talented artists and poets among its victims resulted in the disease being romanticized by some as ennobling. With no effective treatment available, it was a slow and painful disease, characterized by coughing, fever, night sweats, weight loss, and wasting. Contemporary with Keats, a host of other English literary giants believed to have had tuberculosis include Percy Bysshe Shelley, Elizabeth Barrett Browning, and the famed Brontë siblings, Emily, Anne, and Charlotte.

The poignancy of Keats’ rapid decline, his artistic drive and boundless productivity, his extreme physical suffering and lack of effective therapy, and his own awareness of his impending demise are all parts of a tragic tale of 19th Century genius cut short by infectious disease that in the modern era is preventable and treatable. Still, from his suffering and endurance came some of the most luminous poetry in the English language—poems of yearning, mortality, and an intense experience of fleeting beauty.
